# Embracing intrinsic capacity and frailty in healthy ageing research

**DOI:** 10.1016/j.tjfa.2026.100216

**Published:** 2026-10-13

**Authors:** Marco Canevelli, Roger Fielding, Marco Inzitari, Wee Shiong Lim, Emanuele Marzetti, Renuka Visvanathan, Matteo Cesari

**Affiliations:** a Department of Human Neuroscience, Sapienza University, Rome, Italy; b Aging Research Center, Department of Neurobiology, Care Sciences and Society, Karolinska Institutet and Stockholm University, Stockholm, Sweden; c Nutrition, Exercise Physiology and Sarcopenia Laboratory, Jean Mayer USDA Human Nutrition Research Center on Aging, Tufts University, Boston, MA, USA; d Parc Sanitari Pere Virgili and Vall d'Hebron Institute of Research (VHIR), Barcelona, Spain; e Department of Geriatric Medicine, Institute of Geriatrics Active Ageing, Tan Tock Seng Hospital, Singapore; f Geriatric Education & Research Institute, Singapore; g Lee Kong Chian School of Medicine, Nanyang Technological University, Singapore; h Department of Geriatrics, Orthopedics and Rheumatology, Università Cattolica del Sacro Cuore, Rome, Italy; i Fondazione Policlinico Universitario A. Gemelli IRCCS, Rome, Italy; j Adelaide Geriatrics Training and Research with Aged Care Centre (GTRAC), School of Medicine, College of Health, Adelaide University, Adelaide, South Australia, Australia; k Aged and Extended Care Services, The Queen Elizabeth Hospital, Central Adelaide Local Health Network, Adelaide, South Australia, Australia; l Department of Clinical and Community Sciences, University of Milan, Italy

**Keywords:** Frailty, Intrinsic capacity, Healthy ageing, Integrated care

The past few years have been transformative for the *Journal of Frailty & Aging (JFA)*, which was founded in 2012 to gather evidence on both the theoretical and operational definitions of frailty. Today, the journal receives submissions from all regions of the world, including low- and middle-income countries, highlighting how population aging challenges and reshapes traditional models of care globally. The journal attracts submissions from a wide range of medical specialties and healthcare professions, reflecting how the concept of frailty has progressively permeated both clinical practice and the broader scientific community. In recent years, there has been a noticeable increase in contributions that aim to clarify the biological underpinnings of frailty (i.e., links to geroscience) or examine the design, implementation, and evaluation of innovative, integrated care models centered on it (i.e., implying stronger implications for public health and health policies).

Another recent spontaneous shift in JFA’s scope and editorial activity has been the growing number of unsolicited submissions and published papers on intrinsic capacity [[1], [2], [3], [4], [5], [6]]. This trend was clearly reflected in contributions presented at past editions of the International Conference on Frailty and Sarcopenia Research, for which JFA serves as the official journal, which has led to the renaming of the annual event to the “Intrinsic Capacity, Frailty, and Sarcopenia Research” conference [7]. Intrinsic capacity was developed by the World Health Organization (WHO) as part of the framework on healthy ageing [8]. It is defined as the composite of an individual’s physical and mental capacities, and organized into five constituent domains: locomotor, cognitive, psychological, sensory, and vitality [9]. Declines in these domains can be prevented and managed through tailored, evidence-based interventions [10]. Together with supportive environments, these measures can help strengthen functional ability (i.e., the ability to be and do what one has reason to value).

As recently noted in a perspective paper published in *Age and Ageing* [11], intrinsic capacity should not be viewed as an opposing, alternative, or competing paradigm to frailty. In fact, intrinsic capacity emerged within a public health framework as a positive indicator of capacities and reserves, making it especially relevant for prevention and early interventions in primary care and community settings [8]. Frailty, by contrast, was conceived in geriatric medicine as a cumulative clinical measure of the negative aspects of aging (i.e., deficits and vulnerabilities) and is suited to stratifying individual risk, guiding care adaptations, and allocating resources in specialist settings [12]. Far from conflicting, these constructs share broader goals in transforming care for older people: i) moving beyond chronological age as the primary basis for decision-making; ii) shifting from disease-centric to function- and capacity-oriented models of care; and iii) supporting holistic, integrated, person-centered care interventions. They also provide complementary perspectives across the continuum of aging: intrinsic capacity captures early declines in physical and mental capacities, whereas frailty reflects the stage at which the accumulation of deficits translates into increased vulnerability and a need for adapted care. Together, they can inform coordinated clinical and public health strategies for aging: frailty highlights vulnerability and the need for specialized care, while intrinsic capacity provides a structured approach to supporting healthy aging and guiding population-level interventions [11]. Overall, these reflections plausibly explain the spontaneous, gradual shift in focus toward intrinsic capacity in a journal such as the *JFA*, whose originating focus was initially on frailty.

From an editorial perspective, JFA welcomes high-quality contributions that advance understanding of the conceptual and biological foundations of intrinsic capacity; examine its trajectories, determinants, and clinical or prognostic relevance; develop and evaluate care models informed by the construct; and assess their implementation, effectiveness, equity, and sustainability in real-world settings. Particular attention will be given to research clarifying how intrinsic capacity and frailty can be used in complementary ways to inform prevention and management, clinical decision-making, integrated and long-term care, and population-health strategies.

As JFA broadens its editorial scope in this direction, scientific contribution and practical relevance will guide editorial decisions. Studies limited to proposing or validating additional assessment instruments without demonstrating clear conceptual, clinical, public health, or implementation value will generally receive lower priority. Measurement studies addressing consequential questions, including validity across populations, responsiveness, interpretability, feasibility, harmonization, and relevance to decision-making, will remain within scope. Although intrinsic capacity should not be reduced to a single mandatory instrument, greater consistency in its operationalization and reporting is needed to support comparability, evidence synthesis, and implementation. The priority should now be to look beyond measurement itself and focus on how intrinsic capacity across the life-course can inform clinical practice, health-system design, public health action, and the development of person-centered models of care. Research that improves the understanding of how augmentation or maintenance of intrinsic capacity contributes to frailty prevention or the mitigation of adverse health outcomes related to frailty will also be a priority interest for JFA.

The journal will remain open to constructive examination and refinement of the WHO construct. Contributions proposing modifications, additional domains, or alternative operationalizations should provide a clear conceptual rationale and demonstrate scientific or practical added value. At the same time, it is important to recognize that the WHO definition of intrinsic capacity carries normative authority that extends beyond that of individual scientific publications or research groups, having been developed to provide a common conceptual foundation for global policy, health-system strengthening, and the promotion of healthy ageing. Accordingly, JFA will seek to avoid unnecessary fragmentation into overlapping labels or subconstructs that could weaken the holistic and integrative purpose of intrinsic capacity.

Through this editorial approach, the journal aims to support a coherent but evolving field, encouraging innovation while maintaining conceptual clarity and relevance to the health and care of older people.

## Funding sources

This research did not receive any specific grant from funding agencies in the public, commercial, or not-for-profit sectors.

## Declaration of the use of generative AI and AI-assisted technologies in scientific writing and in figures, images and artwork

No generative AI or AI-assisted technology was used in the preparation of this manuscript.

## CRediT authorship contribution statement

**Marco Canevelli:** Conceptualization, Writing – original draft. **Roger Fielding:** Conceptualization, Writing – review & editing. **Marco Inzitari:** Conceptualization, Writing – review & editing. **Wee Shiong Lim:** Conceptualization, Writing – review & editing. **Emanuele Marzetti:** Conceptualization, Writing – original draft. **Renuka Visvanathan:** Conceptualization, Writing – review & editing. **Matteo Cesari:** Conceptualization, Supervision, Writing – review & editing.

## Declaration of competing interest

The authors declare that they have no known competing financial interests or personal relationships that could have appeared to influence the work reported in this paper.
